# Supplementary material for: CD8+ and Regulatory T cells Differentiate Tumor Immune Phenotypes and Predict Survival in Locally Advanced Head and Neck Cancer
Source: Cancers (Basel). 2019 Sep 19;11(9):1398. doi: 10.3390/cancers11091398 (PMC6769847; doi:10.3390/cancers11091398)
Supplement: Supplementary file 1 [file cancers-11-01398-s001.pdf]

Supplementary Materials: CD8+ and Regulatory T Cells Differentiate Tumor Immune Phenotypes and Predict Survival in Locally Advanced Head and Neck Cancer

Alessia Echarti, Markus Hecht, Maike Büttner-Herold, Marlen Haderlein, Arndt Hartmann, Rainer Fietkau and Luitpold Distel

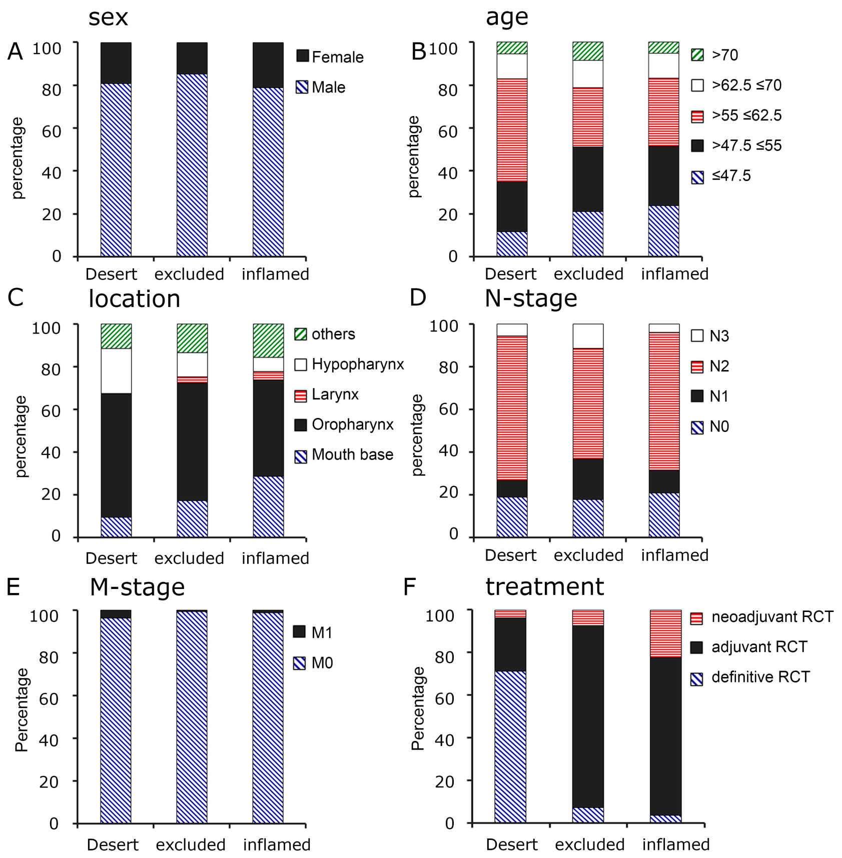

Figure S1. "Immune desert" was defined as having ≤ 50 CD8+ cells in the stromal compartment, "immune excluded" < 500 CD8+ cells in the intraepithelial and > 50 in the stromal compartment and "inflamed" as having > 500 cells in the intraepithelial compartment. Clinical characteristics for patients sex (**A**), age (**B**), tumor location (**C**), regional lymph node metastases (**D**), distant metastases (**E**) and treatment in the three subgroups.

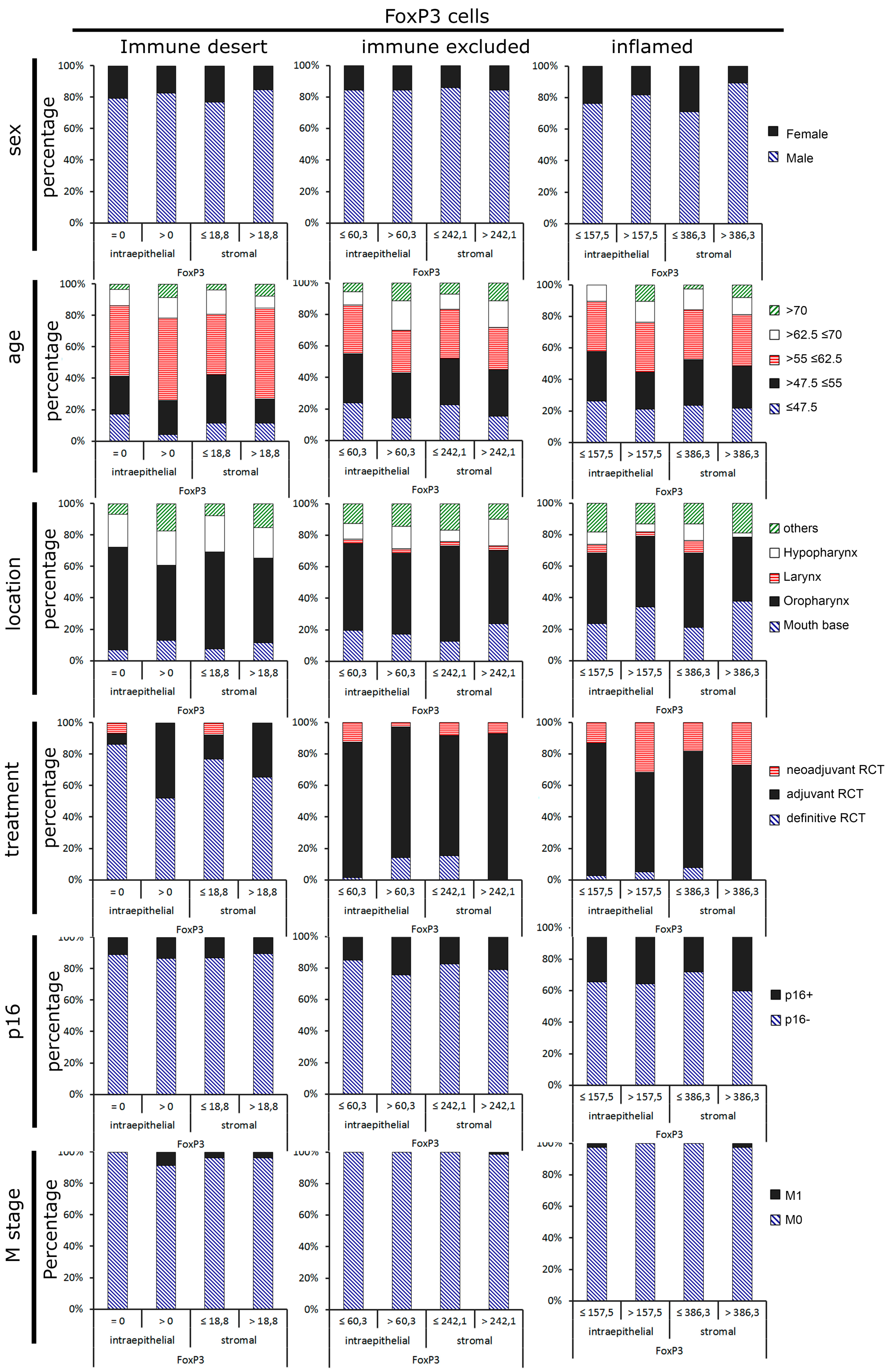

Figure S2. FoxP3+ T lymphocyte densities (cells/mm²) in the intraepithelial and stromal compartment of the "immune desert", "immune excluded" and "inflamed" groups. Tumor characteristics with regard to patients sex (**A**), age (**B**), tumor location (**C**), treatment modalities (**D**), p16 staining (**E**) and distant metastases (**F**) compared in the three subgroups according to FoxP3+ densities. Cut-off values were the median FoxP3+ densities.
